# What Is Old Age? Differences in Views of Old Age Between Employees and the Self-Employed

**DOI:** 10.1177/00914150251401564

**Published:** 2026-01-13

**Authors:** Nabaraj Adhikari, Kasper Kotisaari, Kathrin Komp-Leukkunen, Visa Rantanen

**Affiliations:** 1 Department of Social Science, 4415 School of Engineering Sciences, LUT University, Lappeenranta, Finland

**Keywords:** old age, meaning, employment, self-employment, life course, retirement, Finland

## Abstract

Self-employment is a way for older adults to become active, achieve financial security and contribute to the economy. We explore the meaning of old age from the perspectives of employed and self-employed people. To examine the meaning of old age, 16 employed and self-employed Finns aged 50 and older were interviewed. The sample was stratified according to gender, employment relationship and company size. We employ the life course perspective to examine how choice of work over the life course influences how people think about old age. Employees and self-employed individuals have different perspectives and ways of defining the onset of old age, particularly in terms of the link between retirement and old age. This paper demonstrates that employees and the self-employed view the beginning of old age differently and have different life courses, showing the complex interplay between work and perception of old age.

## Introduction

A rapidly growing ageing population has significantly changed labour market dynamics, and older adults now form a growing segment of the labour market (Kautonen et al., 2017). This demographic shift has led to policy changes that aim to extend working life, which in countries with a state pension schemes thus delays dependency on government resources by delaying exit from the labour market (Caines et al., 2019). Changes in government policy have led to people remaining active beyond retirement age, and self-employment or entrepreneurship is emerging as an important alternative to conventional employment in later life (Kautonen et al., 2017). Self-employment is receiving particular attention from government authorities because it is considered a way to secure financial well-being in later life and to enable older citizens to contribute to the economy (Halvorsen & Morrow-Howell, 2016). Self-employment can be a tool for extending working life, and older adults are encouraged to pursue self-employment later in life. Extending working life allows people to continue working, make career choices, and contribute to society through their valuable knowledge and years of experience. Thus, self-employment is changing our concept of work and the meanings we associate with later life (Halvorsen, 2020).

‘Old’ usually refers to individuals who are nearing the end of their life (Overall, 2016). However, ‘how old is old?’ is a philosophical question. The concept of old age and the ageing process is socially constructed and influenced by the societal perceptions of various age groups (Powell & Hendricks, 2009; Taylor & Earl, 2016). It is generally accepted that employment plays a significant role in identity formation, including identity in old age (Manor, 2017). Kohli (2007) argues that the lives of individuals are structured around work, and retirement is an important event on a person's life course indicating the end of work (Kohli, 2007). When people leave employment or retire, their life routines undergo substantial changes, which has a significant impact on their social, economic and family spheres (Sargent et al., 2013). Therefore, retirement is often considered the beginning of old age (Manor, 2017). For example, Kaufman and Elder Jr (2002) argue that retired individuals often perceive themselves as old and tend to associate retirement with old age.

Denton and Spencer (2009) define retirement as the transition from a work-centred life to a period focused on personal activities and leisure as one enters old age. This transition is clearer for employed individuals, with the official retirement age set by government policies. However, self-employed individuals, particularly those who continue to work beyond retirement, and those who never fully retire, do not have the same distinct transition. Nevertheless, studies have shown that self-employed individuals exhibit distinct retirement patterns (Anxo et al., 2019; Hochguertel, 2015). For example, the self-employed tend to retire later, surpassing the state pension age (Hochguertel, 2015; Komp-Leukkunen, 2025; Nagore García et al., 2021; Zwier et al., 2021) resulting in uncertainty about retirement plans among self-employed individuals (Cobb-Clark and Steven, 2009; Zwier et al., 2021; Polvinen et al., 2024). As a case in point, Wahrendorf et al. (2017) reported that the odds of working beyond the state pension age in Europe are eight times higher among the self-employed compared to those who are employed. Other studies have indicated that in Europe, self-employed individuals retire on average two years later than employees (Hochguertel, 2015). Manor (2017) claims that for employees, retirement is the official exit from the workforce, thus representing definite entry into old age; however, the self-employed might perceive this stage differently, as they continue to work. This continued engagement in meaningful work experiences provides greater social connectedness and cognitive stimulation, leading to a more fulfilling old age. Studies have also indicated that continued employment is positively associated with older adults’ financial security and psychological well-being (Schwingel et al., 2009). Jurek’s (2021) studies also suggest that the higher the retirement age, the later the subjective onset of old age, showing that retirement influences individuals’ self-perception.

The above discussions suggest that employees and self-employed individuals have different perceptions of old age. However, research exploring the differences in meanings associated with old age among employed and self-employed individuals is limited. This paper attempts to fill this knowledge gap and shed light on the different meanings of old age from the perspectives of employed and self-employed individuals. To do so, we ask the following questions: (1) Does the perception of the beginning of old age differ between employees and self-employed individuals? This question probes the idea that old age starts with retirement (Komp, 2017; Manor, 2017), exploring whether it is held by both employees and the self-employed. (2) How do views of old age differ between employees and the self-employed? This question probes the understanding that old age is a time without paid work (Vangen et al., 2021) and attempts to examine how far it resonates with employees and the self-employed. (3) How does gender play in any differences in perception of the beginning of old age? (4) How does company size play in any differences in perception of the beginning of old age? These question draws on well-known differences in retirement behaviour across genders and company sizes, and seek to establish whether they play out differently for employees and the self-employed (Kim et al., 2024; Komp-Leukkunen, 2024).

Addressing these questions will provide valuable insights into the concept of old age in the context of prolonged careers. Thus, this study will help us understand how varying experiences and work modes affect perceived meanings of old age, thereby providing valuable knowledge that can contribute to the formulation of policies to support financial stability and improve quality of life during old age. This study is important in the Finnish context due to its fast-growing ageing population and increasing number of self-employed individuals (Statistics Finland, 2021), which allows for effective investigation of perceptions of old age.

## Old Age From a Life Course Perspective

**Figure 1.**
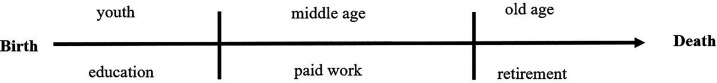
The tripartite life-course model.

In this article, we use Martin Kohli's life course theory as a theoretical framework for examining how people's experiences in their earlier lives shape their views of old age. Studies into life courses indicate that human life is not isolated and is shaped by societal influences (Komp-Leukkunen, 2020; Settersten, 2017). Researchers have argued that the organisation of work and employment plays a central role in shaping person's life course (Kohli, 2007; Komp-Leukkunen, 2013). The tripartite life course model proposed by Kohli provides a structured means of understanding how individual lives are organised in modern societies. This model suggests that an individual's life can be divided into youth, middle age and old age, as shown in Figure 1 (Kohli, 1988, 2007; Komp & Johansson, 2015, 2019).

The three phases of youth, middle age and old age are relatively distinct from one another and can be organised around education, paid work and retirement. Education is the focus of the youth phase, a period of preparation in which individuals learn the skills and knowledge that will be necessary for their future work. In the current social context, this stage is longer than in the past due to labour market demands that require higher levels of education and specialisation (Choi, 2021). The second phase, middle age, is linked to work and characterised by employment, productivity and contribution to the economy. This is the longest phase of an individual's life course, in which they have a clear social role as an economically active worker or professional. The final phase of life, old age, is linked to retirement. It starts when individuals exit the workforce, which is assumed to happen at the time when their health declines (Kohli, 1988, 2007; Komp & Johansson, 2016). In this period, individuals are supported in many countries by the pension and social security system. This establishes a link between the end of paid work and old age and suggests that retirement defines who is old and who is not (Komp-Leukkunen, 2013).

Kohli's framework expects that individuals will follow a linear path throughout these stages, and in such cases, it can be assumed that there is a certain level of predictability and consistency in life trajectories. Thus, individuals following the traditional employment path often experience a more stable and predictable life course trajectory (Wang & Shultz 2010), reflected in regular income-structured retirement plans and organisational support systems such as healthcare and pension benefits.

Consequently, employed individuals experience fewer disruptions and more consistent life planning, resulting in a smoother transition to old age (Wang & Shultz, 2010). On the other hand, the life course of the self-employed is more complex and often deviates from the tripartite model. The boundaries between education, work and retirement are often unclear. Self-employed individuals’ incomes and job security depend on market conditions and their ability to adapt to changing circumstances. For some, self-employment results in financial success, while for others it can lead to periods of financial instability. Self-employed individuals may work beyond traditional retirement age (Hochguertel, 2015) due to a lack of structured support to maintain financial stability, and are responsible for their own taxes, insurance, and success and failure (Gerpott et al., 2021). The differences in life courses between employees and the self-employed reflect inequality in terms of economic and social protections. Employees with stable and long-term jobs receive support from the workplace such as health insurance, unemployment benefits and pensions (O’Brien, 2003), while self-employed individuals do not have access to these benefits, making their financial situation less secure. Self-employed individuals do not receive health insurance from an employer and are responsible for their own insurance (Berkowitz et al., 2021). This lack of support continues into retirement, where self-employed individuals may have less access to retirement plans, leading to greater financial uncertainty in old age. Employees, on the other hand, have clear transitions with a specific retirement age, and these transitions are socially recognised and provide a sense of achievement or sense of closure (Atchley, 1989) that enhances the feeling of old age as a period of rest and leisure. Self-employed individuals might not experience such clear transitions, instead experiencing a gradual reduction in work activities and then gradual retirement (Zissimopoulos & Karoly, 2007). However, self-employment often allows flexibility in work (Hundley, 2001), leading to a more personalised transition. In addition, self-employed individuals have a greater sense of personal agency as they have more control over their career paths, which are driven by personal decisions and efforts (Stephan et al., 2020), whereas employed individuals might feel they are under external control and need to rely on government policies and retirement plans. As such, these varying life trajectories lead to diverse life experiences among employees and the self-employed, resulting in different meanings being attached to later life.

## Old Age After Employment and Self-Employment in Finland

In Finland, the self-employed have distinct retirement patterns and experiences that differ from those of employees. An individual's retirement path is connected to income changes, and their income is affected by pensions and taxes which depend on their retirement path (Riekhoff, 2018). For example, the study on the differences between work and retirement patterns of employees and the self-employed in Finland by Polvinen and colleagues found that employed individuals tend to have more stable income during old age, particularly if they continue to work after retirement (Polvinen et al., 2024). Self-employed individuals however, often have less income stability in old age. This income stability varies according to the type of self-employment. Moreover, 84% of self-employed people in Finland choose to make lower contribution to their pensions insurance, resulting in inadequate protection during old age (Salonen et al., 2020). Finland's Self-employed Person's Pension Act (Yrittäjän eläkelaki) regulates pension coverage for the self-employed, who must make mandatory contributions to pension insurance if their tax-declared income exceeds a certain level and their self-employment continues for more than four months (Finnish Centre for Pensions, 2024). However, self-employed individuals in Finland are free to decide their contribution base, which has an effect on the contributions made and eventually the pension paid. In 2024, the pension-declarable income for self-employed people was from 9,010 (minimum) to 204,625 Euros (maximum) per annum (Finnish Centre for Pensions, 2024). A study by Salonen and colleagues reported that self-employed individuals in Finland often declare minimal income in order to pay less in contributions, resulting in lower pension security than employees (Salonen et al., 2020). In contrast, employees generally experience stable retirement patterns and income security in Finland due to their participation in mandatory pension insurance during their working lives, with contributions automatically deducted from their salaries. In addition, employers in Finland must contribute to a pension scheme where the government sets the contribution rate (OECD, 2021), which further strengthens the pension security of employees, giving them more reliable and predictable incomes during old age.

Employees in Finland normally experience a structured transition into retirement due to the mandatory retirement age, while the self-employed experience a heterogeneous transition. Komp-Leukkunen identifies three main retirement transitions among the self-employed: direct transition from self-employment to pension receipt, self-employment to non-employment to pension receipt, and self-employment to employment to pension receipt (Komp-Leukkunen, 2024). Self-employment allows people to have flexible retirement options and to be in control of their transition (Zwier et al., 2021). Employed individuals, however, face mandatory retirement when they reach the state pension age, and it can therefore be argued that the two groups have different retirement transitions, where the self-employed retire later, and face economic instability due to inconsistent pension contributions.

The pensionable age for females in Finland is 65 for those born in 1965 or earlier, the same as for their male counterparts (Finnish Centre for Pensions, 2024). However, women generally have shorter work histories compared to men, resulting in a notable gender gap in pension income (Bettio et al., 2013). A study found that in Finland women receive, on average, a quarter less pension income than men (Kuivalainen et al., 2020). This difference is similar for self-employed males and females (Kuivalainen et al., 2020). Furthermore, the study highlighted that women are less likely to receive pensions derived solely from their earnings due to their short working careers. For example, 85% of men in Finland receive pensions derived solely from their earnings, whereas only 70% of women do so (Kuivalainen et al., 2020). These findings suggest that females have different economic experiences than males during later life.

As postulated by life course theory, the meanings attached to old age are significantly shaped by the social and structural context, life experiences, and life transitions. Retirement represents a crucial life transition after which the employed and the self-employed have varied experiences. Thus, it can be claimed that old age holds different meanings for the employed and the self-employed in Finland. In this research paper, we aim to explore the life experiences of employees and the self-employed and investigate how these experiences shape their perception of old age.

## The Interplay Between Gender, Workplace and Perceptions of Old Age

Studies have indicated that gender and workplace influence individuals’ perceptions of old age. Women tend to view the onset of old age as earlier than men (Barrett & Von Rohr, 2008), possibly because women tend to retire earlier than men (Edge et al. 2017; Kim et al.). Women also tend to have a more positive image of old age than their male counterparts (Kornadt et al., 2013). Similarly, the retirement transition varies significantly between people who are solo self-employed and those who own a company. For example, Komp's studies comparing how people who work for themselves and those who own businesses transition into retirement show that company owners tend to retire later than sole-trader self-employed people (Komp-Leukkunen, 2024). These discussions suggest that employees and self-employed individuals have different experiences and views of old age depending on their gender and type of organisation.

## Method

### Design

We adopted an explorative qualitative research approach. We invited individuals aged 50 and above who were still employed or self-employed to participate in the study. A total of 16 participants took part in the study. We stratified the sample based on type of employment and company size, first dividing it into employees and the self-employed, and then based on company size (small vs. large workplaces; see Table 1). In the case of self-employed people, the solo self-employed were classified as working in small workplaces, while the self-employed with employees were categorised as working in large workplaces. Companies with less than ten employees were classified as small workplaces, and companies with more than ten employees as large workplaces. Gender balance was maintained within each stratum. Study participants were recruited through advertisements in Facebook groups, using the personal contacts of the research group members, and by approaching entrepreneurs’ organisations. In addition, we sent emails to various workplaces and companies and walked into shops to ask people to volunteer to participate in the study. Individuals employed in the agricultural sector (both self-employed and employees) were excluded due to the distinct nature of entrepreneurial activities in family-owned agricultural businesses (Rautiainen et al., 2019) and the employment of migrant farm workers (Juskaite, 2024).

**Table 1.** Sampling Strata and Characteristics.

Company/employment type	Self-employed				Employees			
	Pseudonym	Sex	Age	Occupation	Pseudonym	Sex	Age	Occupation
*Small workplace*	*Solo self-employed*				*Employee in small workplace*			
	Juhani	Male	59	Musician	Ilmari	Male	52	Architect
	Johannes	Male	65	Architect	Kalevi	Male	60	Restaurant manager
	Lilja	Female	78	Craftsperson/Merchant	Orvokki	Female	58	Salesperson
	Maria	Female	61	Freelance Journalist	Päivi	Female	60	Chef/shift manager
*Large workplace*	*Self-employed with employees*				*Employee in larger workplace*			
	Viljami	Male	56	Software Company CEO	Emilia	Female	58	IT specialist
	Tapio	Male	52	Sports Business Owner	Sofia	Female	60	Senior facilities supervisor
	Kerttu	Female	60	Restaurant Owner	Olavi	Male	51	Researcher
	Linnea	Female	59	CEO/Funeral Home and Florist	Toivo	Male	57	Software designer

### Data Collection and Analysis

Interviews were conducted in Finnish between May 2024 and August 2024 using a semi-structured interview guide with open-ended questions about old age. The semi-structured interview technique allows for flexibility in the interview protocol, as the interviewer can use follow-up questions, and probe comments and responses during the interview, hence facilitating in-depth conversation (Creswell, 2018). This approach enabled the interviewer to explore participants’ thoughts, feelings and experiences related to old age. The interview guide was divided into three sections, namely: (1) ‘their own life’, (2) ‘general views on old age’ and (3) ‘their own old age’. These sections included various open-ended questions on the respective themes. All participants were interviewed for up to an hour, and the interviews were audio-recorded. Seven of the interviews were conducted face-to-face, seven using the Zoom video communication platform, and two by telephone (as shown in Table 2).

**Table 2.** Interview Characteristics.

Interview number and pseudonym	Interview mode	Platform	Length of interview (minutes)	Transcript length (word count)
1. Emilia	Remote	Zoom Video Communication	00:57:38	8724
2. Olavi	Remote	Zoom Video Communication	00:58:07	8654
3. Juhani	Face-to-face	–	00:27:46	3327
4. Johannes	Face-to-face	–	00:25:35	2489
5. Viljami	Remote	Zoom Video Communication	00:24:15	3968
6. Maria	Remote	Zoom Video Communication	00:34:53	5823
7. Sofia	Face-to-face	–	00:31:26	5130
8. Lilja	Face-to-face	–	00:55:24	8310
9. Kerttu	Face-to-face	–	00:48:47	9621
10. Toivo	Remote	Zoom Video Communication	00:49:55	6856
11. Linnea	Face-to-face	–	00:50:16	6831
12. Ilmari	Remote	Zoom Video Communication	01:23:52	15339
13. Orvokki	Remote	Telephone	00:33:18	3903
14. Kalevi	Remote	Zoom Video Communication	01:07:17	10420
15. Tapio	Remote	Zoom Video Communication	00:21:51	3929
16. Päivi	Remote	Telephone	00:48:54	7540

We used a thematic data analysis technique to analyse the interview data. This is a form of content analysis that involves well-defined procedures that allow researchers to identify patterns, themes and meanings within qualitative data and provide a deeper understanding of participants’ perspectives. This approach was deemed suitable for this research because it is an inductive approach designed to extract rich and complex narratives from the data (Braun & Clarke, 2012).

Firstly, the interviews were transcribed in Finnish using the transcription tool ‘Faster-whisper-large-v2’ (SYSTRAN, 2023) based on OpenAI's Whisper speech recognition model (Radford et al., 2023), which was specially designed to transcribe a wide range of languages, including Finnish. It supports offline transcription which prevents data leaks and thus protects sensitive personal information and ensures compliance with the European Union's General Data Protection Regulation (GDPR). After this step a research assistant checked and corrected the automated transcripts to create verbatim transcripts. The data was then coded using qualitative content analysis. Our coding framework was organised according to a data analysis framework structured around the interview guide and research questions. Common themes were identified and categorised through coding, with notable quotes documented to support these findings. The participants’ privacy was protected by maintaining high standards of confidentiality in accordance with GDPR regulations.

## Findings

### Perceived Onset of Old Age According to Employees and the Self-Employed

In this study, the perceived onset of old age means the subjective point in life when employees and the self-employed begin to identify themselves or view others as old. The feeling of being old is a very personal and multifaceted experience influenced by various factors. This study found that employees and the self-employed associate the onset of old age with the following factors: chronological age, retirement, health conditions, functional capabilities, individual milestones, and mindset and perception. Employees and self-employed individuals experience these factors differently, reflecting their different life situations, occupational structure and personal beliefs.

Chronological age is a widely expressed marker of the beginning of old age. Employees and self-employed people used varying ages when describing the beginning of old age. Employees perceive old age to start between 60 and 70, but the feeling of being old depended on health and physical functioning rather than advancing age. For employee, reflection may be triggered between the ages of fifty and sixty by reaching certain milestones.

Sometimes you hear from your own children that you’re already really old [laughs] but… when someone is in their seventies, I think, they already have a few, all sorts of ailments… I can’t say for sure now, but when you are in your seventies, maybe then you are old. (Orvokki, female, 58, employee in a small workplace)

Similarly, self-employed individuals also resist identifying a specific chronological age; they prefer to provide a broader range of ages and often link the concept of old age with health and functional capacities. For them, age 70–80 is the potential threshold when one might start feeling old, but it does not signify old age by itself. They often associate this period with physical and cognitive decline and thus old age.

I would say that above seventy, maybe you can say that people are old. I don’t know, well maybe not… well, for example, my mother is 80, but I couldn’t classify her as elderly under any circumstances. Yeah, well of course she's aged, and her age shows, but in my opinion, it's something that can’t be, like, age can’t be precisely defined by numbers. (Juhani, male, 59, solo self-employed)

This insight demonstrates a common belief that chronological age is insufficient to define the beginning of old age; instead, both employees and the self-employed explain old age from a broader health and functional perspective.

In this study, retirement emerged as a significant factor associated with the beginning of old age. Employees perceived retirement as an institutional and structural marker that indicates the beginning of old age. They expressed retirement as a formal acknowledgement of old age imposed by workplace policies and societal norms and describe retirement as an ‘official stamp’ of being old.

But that's what the pension makes possible […] In a way, it brings that stamp of being old, on your forehead in a certain way, meaning when you’re on an old-age pension, then you are definitely old. (Emilia, female, 58, employee in a larger workplace)

Some employees viewed retirement as a transition to relaxation and freedom, where they can pursue personal interests and hobbies besides work, while some employees linked retirement with symbolic entry to old age.

Probably when I retire; at that point I’ll start to think I am old now. (Päivi, female, 60, employees in a small workplace)

In contrast, self-employed individuals did not view retirement as indicating the beginning of old age and expressed their desire and intent to continue working. They often view retirement as irrelevant to being old, resisted the idea of retirement and view work as an integral part of their life. This difference suggests that occupational structure influences perception of the onset of old age, with employees aligning old age with institutional milestones while the self-employed prioritise autonomy and continuous engagement.

Well, it's not relevant at all. Kind of a bad question in itself. (Johannes, male, 65, architect, solo self-employed)

Both employees and the self-employed highlighted deteriorating health as an indication of old age. Employees see various health conditions such as Alzheimer's and musculoskeletal issues as a clear indication of the beginning of old age, as this impacts a person's ability to work and maintain independence. Similarly, self-employed individuals emphasise health as an essential determinant of the onset of old age as it affects individuals’ functional and cognitive abilities.

It depends on illnesses, so what diseases you have; these can be physical, or can be like memory disorders. I mean, it's not necessarily always age-dependent. But if I had to say a certain age then I would say 80, but it really depends on the ability to function, on the diseases, all those kind of things that affect memory, surely. However, I think memory deteriorates with age for all of us, but when some significant limitations to memory develop, maybe that is old age. (Maria, female, 61, solo self-employed)

Both, employees and the self-employed viewed functional capabilities as a critical marker of the onset of old age. Individuals associated the inability to perform daily tasks, failure to maintain independence, and inability to adapt to life changes with being old. Employees viewed declining physical and mental sharpness as an indication of old age as it affects a person's personal and professional productivity. The self-employed participants had similar views, emphasising functional decline as the beginning of old age rather than associating it with chronological age.

Employees and the self-employed also indicated that various events and transitions which shape one's identity during the life course can trigger the internal realisation of being old; reaching a certain age, participating in someone's birthday celebrations and experiencing physical changes carry social and personal significance and initiate self-evaluation. Interviewees expressed that reaching a certain age, such as fifty, sixty, seventy or eighty, triggers a sense of self-evaluation of past experiences, including life achievements and challenges. Employees experienced this internal realisation between their forties and sixties, whereas self-employed people experienced it when they reach their sixties or even later. For instance, Maria felt that reaching sixty brought a sense of crisis, making her realise the finite nature of life. In contrast, for some individuals reaching these milestones is about reflecting on how they overcame their life challenges and preparing for the remainder of their lives:

Well, I myself didn’t start thinking about it until I turned 60 […] At that point, I suffered a crisis, and from what I’ve talked about with friends, quite a lot say that as long as your age starts with a five, it doesn’t feel like anything, but then when you get to 60, somehow the finite nature of life, it truly hits you. (Maria, female, 61, solo self-employed)

Neither the employees nor the self-employed always linked old age with chronological age; instead, they believe that an individual's mindset and self-perception shape the feeling of being old. In this study, employees and the self-employed acknowledged that old age is a mental construct, meaning that individuals may perceive themselves as old when they personally feel old.

I think it's more like, in a person's own head. Really, if you want to feel old, then you’re old, no matter what age you are. (Sofia, female, 60, employee in a larger workplace)

In this study, a significant proportion of self-employed individuals associated old age with individual mindset instead of chronological age, and this self-perception of being old was related to health and vitality.

It's really inside your head when you are old or, well, age. Of course, inevitably, I am ageing too, but I don’t feel old yet. (Lilja, female, 78, solo self-employed)

Self-employed individuals expressed the feeling of a person being young at heart because they can actively engage in work and life and demonstrate mental flexibility, showing self-employed individuals tend to associate old age with autonomy and mental resilience and view old age as a state of mind rather than a chronological age. They broadly reject institutional markers such as retirement and adopt a more individualised approach to the beginning of old age. In contrast, employees feel old age begins after a significant life event such as retirement.

**Figure 2.**
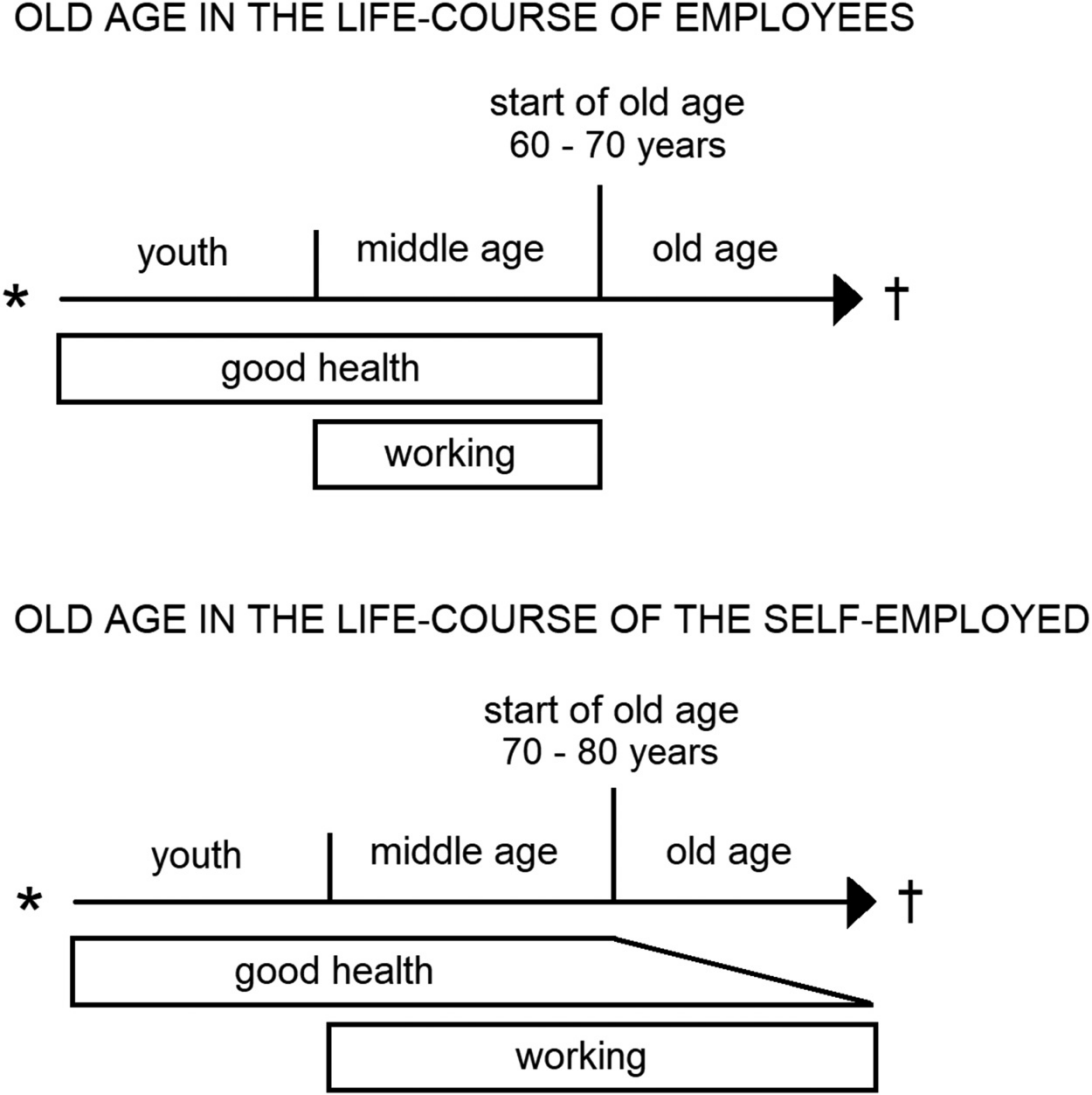
Perceived beginning of old age (difference between employees and self-employed).

These findings suggest that the perceived onset of old age is not merely a chronological number but a complex interplay of structural, health-related and psychological factors. There were notable differences between employees and the self-employed, with employees linking the onset of old age with societal and instructional markers such as retirement which represent formal acknowledgement of old age, while the self-employed associate the onset of old age with more individual subjective experiences rather than external markers, as shown in Figure 2.

### Views of Old Age among Employees and the Self-Employed

In this study, employees and self-employed individuals exhibited diverse views of old age due to their unique life experiences, professional engagement, and the varying degrees of autonomy they enjoy in their professional lives. They have had various positive and negative life experiences. Therefore, they imagine and conceptualise their later lives through these life experiences. Employees and self-employed individuals imagined and associated old age with a period of physical and mental degeneration, diminishing functionality, wisdom and maturity, meaningful moments and proximity to death. There were subtle differences between two groups, with employed individuals tending to view old age from the perspective of post-retirement life and social engagement, while self-employed individuals focused on ability to work, independence and autonomy (Table 3).

**Table 3.** Differences in Views of Old Age Expressed by Employed and Self-Employed Individuals.

View of old age	Employed	Self-employed
Degeneration	Health conditions impact daily routines and job performance. Difficulty maintaining social functioning.	Health issues affect independence and autonomy. Physical and mental health issues affect performance of work tasks and social functioning.
Diminishing functionality	Struggles in social interactions and maintaining hobbies.	Associated sluggishness and fatigue during work tasks. Difficulty pursuing physical hobbies (sports, etc.).
Wisdom and maturity	Associated with calmness, wisdom, self-esteem and self-awareness.	Learning to appreciate life, increase in professionalism and broader life perspective.
Proximity to death	Awareness of death due to the loss of peers or personal health issues. Engage in conversations about death with their social circle and begin preparations.	Awareness of proximity to death but avoid focusing on death. Health conditions make them conscious of mortality.
Defiance of old age	Feel young mentally but acknowledge physical ageing. Resist being labelled ‘old’ (e.g., ‘grumpy granny’).	Feel young due to good health and the ability to work. Accept ageing but resist its negative connotations.
Meaningful moments	Enjoy free time post-retirement. Retirement brings freedom in terms of time and financial resources. Spend more time with family and pursue hobbies. Opportunity to pursue unfulfilled passions.	Flexibility to balance work and personal time. Increased opportunities for family engagement and personal fulfilment.

Degeneration was a prominent theme that emerged in interviewees’ views of old age. Both employees and the self-employed associated old age with deteriorating physical and mental health. They often viewed deteriorating health, such as knee and hip problems, diabetes and hypertension, as disrupting daily life, undermining independence and impacting activity levels. Employees often framed health challenges in relation to work when these health issues affect job performance and daily routines. For example, Kalevi highlighted that his health conditions negatively affect his performance.

I notice about myself, well, I had a myocardial infarction and […] well, I know I’m slower and stiffer than before, things like that you notice, but on the other hand I also notice that […] maybe, yes, people's attitudes change a little bit as I get older. (Kalevi, 60, male, employee in a small workplace)

Self-employed individuals, meanwhile, viewed old age as a challenging life period where people have difficulty maintaining their independence due to physical and mental health issues and are more concerned about their autonomy. For example, Juhani expressed his concerns that health conditions during old age might lead to a loss of physical and cognitive abilities and hinder his individual abilities and social functioning.

I hope that… I won’t suffer from many ailments due to old age that will hinder my life and make my life and that of my loved ones too difficult. Of course, memory disorders are really scary indeed. So yes, you could say that getting older does scare me, but that fear is by no means dominant; well, the downside is, of course, the loss of strength… a decrease in both cognitive and physical abilities, I guess that's probably pretty obvious. (Juhani, male, 59, solo self-employed)

‘Diminishing functionality’ was another shared aspect of how employees and the self-employed view old age. They relate old age to a period when they experience reduced endurance and stamina, impacting their ability to work and their functional capabilities. During old age, individuals’ ability to work or functionality diminishes due to reduced physical capabilities such as impaired mobility, which may further be exacerbated by various health conditions. Many self-employed interviewees linked old age with sluggishness and wear, meaning people become slow while performing tasks during old age and quickly feel physically and mentally drained. Some individuals expressed that they have become more conscious of their performance of specific physical tasks, such as climbing, berry picking, bicycling, and so on, and are also concerned about their physical recovery after performing these tasks. Both employees and the self-employed associate old age with a period where managing household and communal activities will be challenging as their bodies cannot perform with the same agility and speed.

Well, maybe now, lately I’ve been doing more sports, so I’ve noticed that, no, my speed is not the same as it used to be. (Viljam, male, 56, self-employed with employees)

Employees and the self-employed also associated old age with the theme of ‘proximity to death’, which carries profound emotional weight. People relate old age with becoming aware or realising that they are close to death. This realisation of the proximity of death is closely linked to deteriorating health or physical decline. Employees linked this to social and shared experiences, such as losing someone in one's social circle, which served as a reminder of morality.

So, in a way mortality comes to you, it starts to feel close at this age because you start to have colleagues who go [die] because of some seizure, and leave your circle. I just had a class reunion over the weekend, and of the sixty of us who graduated, thirty were there. Of the sixty, two have departed, have gone into eternity. (Emilia, female, 58, employee in a larger workplace)

For the self-employed interviewees, the realisation of the proximity of death was introspective and personalised. This realisation evolved gradually, and they associated old age as a preparatory phase for death.

You physically deteriorate, and kind of like, well, your cognition and memory, all those kinds of things work a little slower and, truly, death is getting closer, it is a fact, and it's just awful. (Maria, female, 61, solo self-employed)

Despite associating old age with negative experiences and challenges, both employees and the self-employed also tended to view old age positively by distancing themselves from its negative connotations, as represented by the theme ‘Defiance of old age’. They acknowledge their ageing process but denied the subjective feeling of being old, as illustrated in the following quote:

Well, I would prefer not to age, of course, but evolution does its job. (Robert, 52, male, self-employed with employees)

This quote presents ageing as a natural and inevitable process, but one which people do not want to view as an old person or experience. Many employees reflected on the individual tension between their physical situation and their cognitive age, and framed their ageing experience in terms of mental youthfulness, emphasising their enduring passion for learning and professional engagement.

Maybe it's just like I said, I feel like I still have the soul of a forty-year-old inside this old body, so I notice my age only because of this body. Due to the fact that I no longer… I can no longer do the things I did 20 years ago or 40 years ago, and that's kind of annoying in a way. (Emilia, female, 58, employee in a larger workplace)

Both employees and the self-employed tended to challenge negative stereotypes, highlighting old age as a period that brings opportunities and positive experiences. The theme of ‘meaningful moments’ showcases the positive aspects and aspirations related to old age; old age is a period for embracing freedom, pursuing unfulfilled passions and personal interests, and enjoying and maintaining family and social connections, often accompanied by retirement or reduced professional responsibilities. Employees linked this freedom with retirement, which brings time and financial resources, allowing them to exercise their autonomy and enjoy life without any fixed schedule.

Well, the good thing is that you don’t have to go anywhere in the morning anymore, and you can lead a relaxed life like that. (Ilmari, male, 52, employee in a small workplace)

They also expressed that old age means fewer financial obligations and family responsibilities, allowing them to focus on self-fulfilling activities.

Well, yeah, certainly, travelling… If I can, financially, then I would like to travel, and of course, going to this island, to the summer cottage in summertime is something I want to do as long as I can. I like to do handicrafts, read books, take exercise as a hobby, cycling, skiing, walking, the gym; I do hope that I will be able to do such things. (Päivi, female, 60, employee in a small workplace)

Similarly, self-employed individuals accustomed to managing their own time view old age as an extension of their independence, a time when they can pursue activities that bring joy and satisfaction to their lives.

I’d like to write a book, this is how I kind of think, that when I retire or when I reduce the amount I work, I’d like to write an autofiction book. (Maria, female, 61, solo self-employed)

Another positive aspect of old age is that it brings ‘wisdom and maturity’. Both employees and the self-employed felt that they will develop insight and become more aware and conscious of themselves and their world due to self-reflection and self-realisation.

Somehow, learn to appreciate life… you learn to maybe enjoy life more, in such a way that you’re not constantly amassing more new experiences, for example, you realise that those experiences, those everyday experiences can be valuable and […] you can somehow, more objectively, deal with the things that life brings. (Juhani, male, 59, solo self-employed)

This wisdom gained through various life and professional experiences often translates into a broader perspective, bringing a sense of self-acceptance, self-confidence and calmness. In addition, people become more appreciative of everyday moments and relationships and approach life with a balanced perspective.

### Views of Ageing and the Onset of Old Age From a Gender and Workplace Perspective

In this study, we observed differences between male and female participants in the perception of when old age begins. The data indicate that female participants were more likely to associate retirement with the onset of old age compared to their male counterparts.

Well, they have already retired a while ago and are celebrating… and this is part of old age. (Orvokki, female, 58, employee in a small workplace)

These finding suggests that females view old age as an institutional transition rather than merely an individual experience, and view retirement as official acknowledgement of that transition and regard it as a time for celebration. In contrast, male participants, especially the self-employed, tend not to regard retirement as the beginning of old age, rather they associate old age with health and functionality. We did not find any significant difference between different company sizes in terms of views regarding the onset of old age; in both small and larger workplaces, individuals generally related old age to functionality and health.

## Discussion

In this study, the first question we aimed to answer was: Does perception of the beginning of old age differ between employees and self-employed individuals? Through this question we aimed to understand the viewpoints of employees and self-employed individuals regarding the idea that retirement marks the beginning of old age. Perceptions about the beginning of old age varied between employees and the self-employed and were linked with various factors such as health conditions, functional capabilities and retirement. Employees tended to consider old age as beginning between the ages of 60 and 70, whereas self-employed individuals resisted indicating a specific age. When they did specify a particular age, they saw old age as beginning later than employed individuals (70–80). In addition, both employees and the self-employed discussed various milestones in their lives or in the lives of others that trigger realisation of the beginning of old age. For many employees, retirement represents a significant structural milestone or indicator of the beginning of old age and the transition away from active work life. This aligns with Kohli's argument that retirement signifies the end of active work life and the start of a new phase in life characterised by freedom from work obligations (Kohli, 2007). Other studies have similarly found that retirement is a marker of old age (Jurek, 2021; Manor, 2017; Wettstein et al., 2024). Sargent and colleagues argued that retirement is a socially constructed concept, indicating that the transition to old age for those following traditional retirement norms (Sargent et al., 2013), which aligns with our findings. Jurek's studies found that the higher the retirement age the later the subjective onset of old age, showing that retirement is an important anchoring factor people use to decide when old age begins (Jurek, 2021); this might explain our finding that the self-employed view old age as beginning later than employees do. Self-employed individuals tend to dismiss the idea of retirement as a marker of old age. For them, the beginning of old age is related to functionality and health conditions, suggesting that they view retirement as less binding with regard to the onset of old age. Consequently, retirement does not hold the same significance for self-employed individuals in terms of marking the beginning of old age. Self-employed individuals have less institutionalised work trajectories, meaning retirement is not a fixed endpoint and old age is more influenced by health and functional capabilities. This highlights Kohli's views on the role of institutional norms in life-course transitions; employees operate within a structured retirement framework (Kohli, 1988, 2007) while self-employed individuals have more individualised pathways. Thus, it can be argued that work plays a vital role in identity formation and changes in work or one's role can determine how individuals perceive themselves (Manor, 2017). Our findings show that a change in individual role from worker to retiree is important for employees to identify as old. In contrast, staying active is a way to resist ageing and create a feeling of staying young (Sargent et al., 2013).

The second question we aimed to address was: How do views of old age differ between employees and self-employed individuals? Through this question, we sought to examine the concept of old age in the context of its relationship to time without work and how employees and self-employed people view this concept and imagine this phase of life. We observed slight variations in views of old age, with employees often perceiving old age as a period associated with a decline in functional capacity, which means the ability to work decreases. Employees also relate old age to retirement, a time without paid work; i.e., they see old age as an institutional shift marked by withdrawal from the labour market after retirement age. This period without paid work is an opportunity for relaxation, enjoyment of freedom, and pursuit of unfulfilled desires and personal interests. For example, employees were excited about the flexibility and freedom retirement will bring, as it will give them time to spend with their families and pursue their interests. Again, this aligns with the tripartite life course model, explaining how different life course stages are experienced (Kohli, 1988, 2007). However, employees are also concerned about health issues and declining abilities, thus viewing old age as a period of preparation for death as people get closer to the end of their life. In contrast, self-employed individuals are willing to continue working if their health allows. For this group, work is important to their identity and purpose. They are keen to express their control over their work. During old age, they will be able to balance their work and their passions and want to continue working if their health allows. Retirement is irrelevant to entrepreneurs, as they can work longer and have the flexibility to do so, showing that for them old age is more a personal than an institutional shift. Therefore, it can be argued that employees tend to view old age as the period of life when the obligation to work has passed and they can pursue their unfulfilled hobbies and desires, while the self-employed want to continue working if their health allows, reflecting the importance of work in their lives. Various prior studies have also found that self-employed individuals view old age as a period of purpose and productivity (Halvorsen & Morrow-Howell, 2016; Sargent et al., 2013; Zwier et al., 2021). They can continue to be involved and engage in work and do not completely exit the labour market, indicating that the later life course of self-employed people differs from the life course model suggested by Kohli. Furthermore, our study found that self-employed people focus on flexibility, job autonomy, and sufficient health to continue working when discussing their views of old age. This aligns with Anxo and colleagues’ findings that individuals with higher job autonomy have better health, and self-employed individuals tend to delay their exit from the labour market (Anxo et al., 2019).

Our third and fourth research questions were: How does gender influence people's perceptions of when old age begins and their views of old age?, How does company size influence people's perceptions of when old age begins and their views of old age? The results indicate gender differences in beliefs about the beginning of old age and views of old age. Female participants tended to associate retirement with the beginning of old age more than their male counterparts, suggesting that females see old age as an institutional shift rather than an individual experience. Female employees view retirement as an official stamp of old age and see it as a time for celebration. In contrast, male participants – primarily the self-employed – see retirement as irrelevant to being old and associate old age with functionality and health conditions. However, we found no clear indication that company size significantly impacts the perception of when old age begins or views of old age. Across both smaller and larger workplaces, individuals generally associated old age with functionality and health.

From the above discussion, it can be concluded that employees and self-employed individuals have varying perspectives on and ways of defining old age and its onset, and that the two groups have different views on the link between retirement and old age. Employees often associate retirement with old age and see it as a period marked by the end of work. In contrast, self-employed people are more reluctant to specify an age at which old age begins, often linking old age to an individual's health and functional ability rather than their chronological age. For self-employed individuals, retirement is not a definitive marker of old age as they can continue to engage in work, resulting in a flexible view of old age. For them, health and physical functioning are the key factors in the decision to continue working rather than institutional norms. Retirement is a personal shift rather than an institutional one, as they can remain involved in work and balance their work with their interests. For employees, old age is a period without paid work and a time for freedom, relaxation and personal fulfilment. They expressed concern about their health during this period and how it will affect their involvement in self-fulfilling activities. These findings suggest that employees and self-employed individuals have different life courses, with employees looking forward to their free time after retirement while self-employed individuals wish to continue working if their health permits. Therefore, flexible policies that allow individuals to work if they are capable are crucial for extending working lives and enhancing productivity later in life.

One of the shortcomings of this study is the qualitative study design, which means the findings are not generalisable to other contexts. In addition, this study was conducted in Finland where employees and self-employed individuals make mandatory pension contributions of varying amounts. This policy context might have influenced the results, particularly the minimal differences in views of old age between employed and self-employed individuals. A strength of this paper is that we examined views and meanings associated with old age from the perspectives of employed and self-employed people, thus contributing unique insights into how people understand later life.

## Conclusion

The perception of old age is a subjective and multifaceted concept; employees tend to link old age to retirement while self-employed individuals focus on health and functionality. This finding suggests that employees and the self-employed view the beginning of old age differently and follow different life courses, highlighting the complex interplay between work and the perception of old age. The findings also suggest that promoting health and functionality can extend the working life of older workers, highlighting that flexible work structures and a positive societal narrative of old age as a period of opportunity and continued contribution can encourage lifelong productivity. This paper also shows that old age is a concept influenced by various personal, social and occupational factors, therefore challenging traditional ideas of old age and emphasising the need for research on ageing to include societal and functional aspects as well as chronological aspects.

## Supplemental Material

sj-docx-1-ahd-10.1177_00914150251401564 - Supplemental material for What Is Old Age? Differences in Views of Old Age Between Employees and the Self-Employed Supplemental material, sj-docx-1-ahd-10.1177_00914150251401564 for What Is Old Age? Differences in Views of Old Age Between Employees and the Self-Employed by Nabaraj Adhikari, Kasper Kotisaari, Kathrin Komp-Leukkunen and Visa Rantanen in The International Journal of Aging and Human Development
