# Supplementary material for: What Is Old Age? Differences in Views of Old Age Between Employees and the Self-Employed
Source: Int J Aging Hum Dev. 2026 Jan 13;103(2):182–207. doi: 10.1177/00914150251401564 (PMC13447798; doi:10.1177/00914150251401564)
Supplement: Supplementary file 1 [file sj-docx-1-ahd-10.1177_00914150251401564.docx]

**Annex I: Themes and Codes**

| **S.N.** | **Theme** | **Name** | **Emp. type** | **Gender** | **Workplace** | **Quotes** |
| --- | --- | --- | --- | --- | --- | --- |
| **Perceived start of old age** | | | | | | |
| 1 | **Chronological age and ambiguity or uncertainty** | Emilia | Employee | F | Bigger Workplace | “Well, I do think at this, at this point, I'm truly starting to say that a person is not old until they are 70 years old. My mom is 87 and she's still doing quite well for her age. She's old, of course, but I'd say, indeed, that my concept of age has changed over time. So, when I was young, then a 50-year-old has been old. |
|  |  | Orvokki | Employee | F | Small workplace | “Sometimes you hear it from your own children that you're already so old [laughs] but uhm, uh, would they, in their seventies, I think, already, old there are already few, all sorts of ailments, ailments, and, no, I can't say more now, there are already seventies, maybe old” |
|  |  | Toivo | Employee | M | Bigger Workplace | “Let's say that it has changed as I myself , remembers my own childhood so, then it feels like back then, someone who turned 50 if he was, grandpa or something like that, like this, yes, felt, old, and maybe that in the 70's somewhere, life was different, then we were already kind of like, we were already kind of like, maybe the last years, at work and work was differently consuming so, then felt but now is ... Let's start going there, maybe closer to seventy, then we'll start to be already, old and then, well, I think yes, you can like, you can do it just fine to seventy depends of course on the task it's not, physically draining or, like, mentally stressful so, closer to seventy if there's anything else, otherwise like, health okay so can but, still be working and, sure, I'm not saying that one who feels old if you start approaching there, between 65 and 70, can still be there, in lighter work, and in that way like that, yes” |
|  |  | Maria | Self-employed | F | Small Workplace | “Who is aged or old, well I mean... in my opinion, if I have to say some age, so aged or old is someone, who is in their eighties. Yeah, it means that and then that certain age, that is, something like eighty so, then I do think you can say that someone is old, even if they don't have limitations in their ability to function.” |
|  |  | Kerttu | Self-employed | F | Bigger Workplace | “I could think that an old, that is, an elderly person, yes, well, maybe like, someone 75 years old, would already be off work in such a way that no, you’d think you'd be like, at least, retire. But, somehow, 80 years old, yes, then it starts to be like that old, but, necessarily, it depends so much on the person.” |
|  |  | Tapio | Self-employed | M | Bigger Workplace | “I guess that then, when, you retire when you retire, so then maybe you can already say that you are a little older, but I don't know now whether the pensioners are yet old either... so yeah, you have to, you have to be over seventy already my own mother is 85 years old, and she is still in very good shape so she drives a car and goes fishing and everything she picks up berries and mushrooms so… of course in terms of age she could be, she could be already aged and, old by now but, otherwise she still is.. in many ways, no one does not think of her as an elderly person, so that let's say that over seventy is like this that then, of course, you can already, think that you would already be, a bit older person.” |
|  |  | Juhani | Self-employed | M | Small Workplace | “I would say that after seventy, maybe you can say that people are [old], say that uhm, I don't know what, well maybe not... well for example, my mother is 80 I, cannot classify her as elderly under any circumstances, Yeah, but, of course, she's aged, and, age shows, in her, so on but, but in my opinion, it's something that can't be like, age can't be, precisely defined, by numbers.” |
|  |  | Päivi | Employee | F | Small Workplace | “I think I will go already there into the seventies so that at least now that I've just turned sixty, so I don't feel myself old nor [laughs] age; well, at least I don't experience it in any way I don't think too badly now [about it], let's say that when I turned fifty so then, I experienced these things... I experienced these things much more intensely... so this milestone of sixty doesn't feel like that... bad... and I don't think about it.” |
|  |  | Linnea | Self-employed | F | Bigger Workplace | “I've always thought that I'll probably be terribly old when I'm in my seventies if I live even that far, but these are our funeral parlour tales [laughs]. Well yeah, maybe it's seventy-, seventy-five so that if I'm, it depends so much on your condition that, how you'll feel yourself old.” |
|  |  | Kerttu | Self-employed | F | Bigger Workplace | Well, yes, I think that when it's eighty, it's going to be like that, then I guess that, now that I always count myself, it's like this, the grief when you think that 61 is so, will come this autumn so, I think that 20 years then I'll be 80 that's the old one. But, I don't yet know what I'm like in my eighties, that I know, 90 over, people who are quite vibrant and refreshed that, but I would think that 80 is starting to be in such a way that then, then it's just that it's kind of like you need that help at least with cleaning or something, things. |
|  |  | Maria | Self-employed | F | Bigger Workplace | “Well, of course I hope never, [laughs]; I guess I'll probably be old in my eighties then.” |
| **2.** | **Mindset and individual perception and perspective** | Tapio | Self-employed | M | Small Workplace | “I've noticed that nowadays so, well, there's, some, since my circle of friends is [so that] almost all of them are entrepreneurs so, some who have been, in business life and, still are and their age is 80 so they are sharper than some, some who are in their fifties so that's kind of, a pretty case-by-case thing so that surely there, there in that uhm, within the older people so, there is so much experience and know-how too that there's sure uhm, I don't really see it so that, I think a person is not if only their mind is functioning then they are not old at any point.” |
|  |  | Linnea | Self-employed | F | Bigger Workplace | “Well, I'd, think it's probably something around 80 is this kind of a milestone that, then you start to, others, of course, it's because of illness if there is, some disease, so, then, of course, it is.. now if we think that we rule out that so that you're like not sick in any way, the person in their 80's is already like… quite old, well a person in their nineties, we, sometimes have people who are, 95 and, may still arrive with their own car. And, 100 years old well maybe not 100 years old but 95; this one granny told me she's doesn't even dare to say how old she is [laughs] “ She was over ninety” |
|  |  | Juhani | Self-employed | M | Small Workplace | “Well, I'd, I would think these days that it starts with seventy… my mother... turned 80 recently, and she still does… works, occasionally so, I do feel that it's pretty much an attitude, an attitude as well, like how and in which way you take care of yourself and how you relate to attitude towards life, in general terms.” |
|  |  | Sofia | Employee | F | Bigger Workplace | “I think it's more like, in a person's own head. Really, if you want to feel old, then you're old, no matter what age you are.” |
|  |  | Lilja | Self-employed |  |  | At least not in age in years [context: meaning you can't decide it by age in years]. It's so much inside your head this, that are you or are you old or, well, aged of course inevitably I am too but, I don't feel old yet |
| **3.** | **Health condition** | Linnea | Self-employed | F | Bigger Workplace | “Well, I would think it's probably something around 80 is this kind of a milestone that, then you start to, others, of course, it's because of illness if there is, some disease, so, then, of course, it is... now if we think that we rule out that so that you're like not sick in any way, the person in their 80's is already like... quite old, well a person in their nineties, we, sometimes have people who are, 95 and, may still arrive with their car …from the general appearance, from everything so that like, and then of course that already, I of course, recognise, if someone has like, poor memory or someone like my mother was, she had Alzheimer's disease for a long time,…and then passed pretty fast then in the end when she ended up in a treatment facility so.. so uhm, I recognise those traits because of that experience” |
|  |  | Maria | Self-employed | F | Small Workplace | “It depends on illnesses, so what diseases you have can be physical, or those can be like memory disorders, so it does. It's not; I mean, it's not necessarily always age-depending. But if I had to say some age, then I say 80, but then it really is so that the ability to function, the diseases, all that kind of things affect, surely then, regarding memory, but for all of us, I think memory deteriorates with age but then that there might come such significant, limitations to the memory and, uhm maybe it is old age.” |
|  |  | Kerttu | Self-employed | F | Bigger Workplace | “That there will be age, of course, but that who, who, whoever is in what condition, will determine quite a lot than that, that is, even a young person can be old, and otherwise, if you are careless, your health is not good” |
|  |  | Toivo | Employee | M | Bigger Workplace | “Of course, from all sorts of illnesses brought on by old age, and even medication, I have had to take blood pressure medication and things like that, so know that it is not uhm, anymore…. unable to eat anything and however much, everything unhealthy” |
|  |  | Toivo | Employee | M | Bigger Workplace | “Well, I always say it's just stupid that a person gets old so no, I think age is , in that way, , I don't like to think about it all the time that this is getting old, but that if you get just, some ailments, that you notice that, you knock about something or, just that you don't bend anymore, the same things as before, so , It's kind of unfortunate, that as it comes, limitations, yes, because they then terribly limit life and, then even if they don't have their own life of course when you remember anymore but then it's like loved ones and, I've seen it that it's terrible, even with Alzheimer's so it's terribly, hard , like close people so , listens like hearing, finds that there is hearing loss- a little, like lowered that it is not like, in some situation when there are a lot of people, around you don't like, children all talk something, then you can't, concentrate that you don't, when you're younger, you can't, register, all the things around, around, but now you have to kind of focus on someone, talking that you, like, get the most out of it when if there's a commotion around, it's just like, you get stuck in such a way that you can't register so many things anymore, like, at the same time” |
|  |  | Emilia | Employee | F | Bigger Workplace | “I won't not before that, and, for now, there are no symptoms, so I haven't investigated the matter. But so that there's in the background just this that, I mean my dad was as well... Dad was a sportsman, sports director, has always moved a lot. And then, when he was a little bit, a little bit over 60 and his mobility started to get worse, he didn't; first, it was probably skiing where he realised that he couldn't ski anymore or couldn't stay standing on the track and, then, all of a sudden he couldn't swim anymore and, like these kinds of things so... so that's when I realised... I was then a little over thirty, about thirty-five years old, so then... I- it was quite a shock to understand that you can't necessarily influence what condition you are in”. |
|  |  | Maria | Self-employed | F | Small Workplace | I believe that, or well, there are different kinds of people in entrepreneurs as well so that, that, [hh] some people continue as entrepreneurs then like, as long as possible and some then again, start to slow down their tasks already at this point in their sixties and then like, [hh] in their own, life, the retiring is not, at all like essential, i- it doesn't that much [] so that okay I kind of think about it, but, if things go the way I want them to, it won't be anything like a single blow but instead, little by little I'll probably then somewhere, around the age of 67 slowly start to reduce those work tasks, or surely their amount will probably decrease already before that. But I do still think now that still for the next years, I'll try to push as, as fully as I can, and then.. maybe there at 65 could it be so that then little by little maybe a little bit less and, and then little by little, a little bit less but, yeah.. so that if someone were to ask me when I'm going to retire so in my opinion it's, a little bit kind of a weird question so that, I haven't like in that way, thought about it but I know that for many people it's like a very important, milestone in life then. |
| **4** | **Functional Capability** | Linnea | Self-employed | F | Bigger Workplace | “So, age is kind of very elusive when you’re in this kind of customer service situation, so that, according to the stream of thought [meaning here: the ability to think or fastness of thinking of that person], my perception of that person's age is actually determined… It might be, so.. because it's, some people might be like in their nineties, they come to buy, maybe a coffin, for a sister who was 88, and, they have such a sharp mind everything and, the talk comes so sharply from there and everything is, just like, nothing and that coffin selling situation is kind of like that it's like, it's like that it's somewhat confusing, for some people”. |
|  |  | Viljam | Self-employed | M | Bigger Workplace | “Maybe it's whether you can, like, master broad entities, like, in conversations... are you able to learn something new when the situation changes, or do you want to get stuck in those, old ones so that you don't do anything, don't do it in a new way you continue in that old way” |
|  |  | Juhani | Self-employed | M | Small Workplace | “So, it's like when I've gotten older, I've noticed that the kind of things that, that you've thought when you were younger that you can't do when you get old-, can't do when you age so it's. it's about nothing else than the fact that people have a habit, a habit of stopping doing something when you get old- older, for example, a certain form of exercise” |
|  |  | Maria | Self-employed | F | Small Workplace | “It depends on the individual so that, so that I think it depends a lot on a person's ability to function, that, what kind of functional capacity they have and that level of activity, and then also on, what they are like doing so that someone can really be like... old, already in their seventies and then, of course, it depends on illnesses so that what diseases, you have so that they can be physical or then they can be like memory disorders, so it does... it's not; I mean, it's not necessarily always age-depending. But if I had to say some age then I say 80, but then it really is so that the ability to function.” |
|  |  | Päivi | Employee | F | Small Workplace | “To be old, well, maybe the fact that it would have, that it already has some effect, in some respect, that it limits certain things. Then you may be old, and if you are no longer able to do everything you did when you were younger, for example, some hobbies, you have had to give up some hobbies so that you are no longer able to do, so then you would maybe already be old”. |
| **5** | **Retirement** | Päivi | Employee | F | Small Workplace | “Probably then when I retire, so at that point I start to think am I old now” |
|  |  | Emilia | Employee | F | Bigger Workplace | “But that's what the pension makes possible, but the pension probably also brings, because then we're really talking about old-age pension, so in a way, it brings that stamp of being old, on your forehead in a certain way, so that when you're on an old-age pension, then you are supposed to be old.”. |
|  |  | Orvokki | Employee | F | Small Workplace | “Well, they already have, retired a while ago and are celebrating….and this belongs to old age.” |
|  |  | Kalevi | Employee | M | Small Workplace | “ Well, that's in itself that you can retire before you've gotten too old and, by the way, but that no, it's exactly, it's like, that old age is like that now, yes, in a way, you can retire before, yes, or but it's worth, I say it's worth retiring before you've gotten too old” |
|  |  | Sofia | Employee | F | Bigger Workplace | “I don't know if it depends on it at all; after all, someone can be old before they retire; it doesn't say that you're old only when you're retired; many people may be old before” |
|  |  | Juhani | Self-employed | M | Small Workplace | “Yes, well, it is, for most people, it's, like in our society, clearly a… very important watershed moment so that it marks the beginning of a whole new phase of life, so but, I, don't see that being the case in my case.” |
|  |  | Tapio | Self-employed | M | Small Workplace | “Well, really again this, old age and retirement, so I have noticed that those friends who, entrepreneur friends who have stayed involved in entrepreneurial life, they are markedly, sharper and more youthful than those, who have now taken that early retirement or something else so most likely I, myself will never retire so that, if it is not this company then it's uhm some other company I guess I'm working on, as long as, uhm, let's say, health and, motor skills give way for it”. |
|  |  | John | Self-employed | M | Small Workplace | “Well, it's not relevant at all. A bit of a bad question in itself.” |
|  |  | Toivo | Employee | M | Bigger Workplace | Well that.. So well not that.. still of course.. can do just that [hh].. date or year to say, but when we start to approach it, the retirement age, of course, if it is possible, then we will start to have, whether it is now for me at the moment, somewhere in sixty, a little before sixty-six, I guess, at the moment it is the lowest retirement age.Well, it starts there, let's say it [hh] about it, maybe it's not like it's a binary thing, that it starts like zero, is between zero and one, but then it starts, |
|  |  | Linnea | Self-employed | F | Bigger Workplace | I guess it's probably, probably a place for people to like, stop that hey, that now I'm pretty old, already. Others will continue in such a way that they, then, they are so, lively and they will go to all the workouts and humppa-dances and so that... they like kind of are even maybe afraid of getting old, I feel like, a lot of people, might be afraid, that now, now that, I've retired, now then, soon, I'm about to lose fit and, but on the other hand it's good that you stay active , at that point. (Linnea, female 59, Self-employed with employees). |
| **6** | **Milestones and realization** | Tapio | Self-employed | M | Bigger Workplace | “Well, not really, not much except at the point when I turned fifty. Well, I was pretty exasperated about it; at that point, I realized that now we're halfway there and, we've crossed the halfway point, so it was just like actually so, but it's okay, sure there I got a little bit, kind of like depressed but it was, it probably lasted for a while but, the facts have to be accepted.” |
|  |  | Maria | Self-employed | F | Small Workplace | “Well, I myself started thinking about it not until I've turned 60, so surely that 60 has clearly been like this kind of, at that point, there was a crisis, and from what I've talked about it like with friends, so, awfully many say that as long as your age starts with a five, it doesn't feel like anything but then when you get to 60 so then, somehow the finite nature of life, it truly like, strikes, and when you think about, the 10 years that how quickly the past ten years have gone from fifty to sixty so then when you think that then the passage of time just speeds up as you get older so that soon there you'll actually be 70, and then there will actually soon be 80 so that it's, that 60 is, in my experience a quite a critical limit so that then you start thinking about it” |
|  |  | Päivi | Employee | F | Small Workplace | “I had this weird experience where, my the fifty years of my life, went, quickly passed me by as diapositives , it, happened like, it happened, somehow so that life revolved [meaning: went on] there and, then I stared at this little stunted pine tree and, at that point when I turned fifty it was also a pretty difficult, difficult phase, phase in my life so, I realized that this stunted, pine has withstood all the storms of the world, and this bedrock has not moved anywhere from here either, no matter what kind of storm has come, so, I decided then that, I, too, I will endure, that I will survive this life... So, this kind of this kind of situation happened then, but, so, now that I became, turned sixty, there was no diafilms playing in my head or anything” |
|  |  | Juhani | Self-employed | M | Small Workplace | “It's a tricky question because it's so that, as I said, changes happen little by little. For some reason a number of years, such as 85 came to mind. Maybe it's such [a number] that, that... when I'm older than that it's like, I at least, don't want to... although I'm a musician I don't, I maybe don't want to bother others any longer with my own playing so that I will have, by then already stopped playing, if I live, that long, or at least I've stopped public performing. Yes, and I suppose that, that by then... also my physical condition will have changed in such a way that, if I'm alive, it's maybe better to lead a pretty peaceful life then, of which you might then say that you are old.” |
|  |  | Sofia | Employee | F | Bigger Workplace | “I guess sometime in my forties when I really had to calculate what [laughing] age you are.. You never thought about it, never like you turned years old. Everyone else always met me, I haven't partied at all, and then, horrible tastes, then woke up sometime in my forties to some damn query when I had to calculate how old you are, oh my god! Am I forty? That like, that, the realisation that, uh, realized that you're already in your forties, yes, it was like, I'm old!” |
| **Image of old age** | | | | | | |
| **1.** | **Degeneration** | Juhani | Self-employed | M | Small Workplace | “I hope, uhm, I hope that... there wouldn't be such ailments due to old age that would hinder one's own life and make one's own life and that of one's loved ones far too difficult. Of course, memory disorders are really scary indeed. So yes, Let’s say that getting older does scare me, but that fear is by no means dominant; well, the downside is, of course, the loss of strength, uhm ... a decrease in both cognitive and physical abilities, I guess that's probably pretty obvious.” |
|  |  | Olavi | Employee | M | Bigger Workplace | “The downside is definitely that one's ability to function and performance deteriorates a bit, I go to the gym 4 or 5 times a week and I keep, track of my results by keeping a gym diary about every day when I go there and then I see these long trends, I can always take a look from there so, performance deteriorates a little bit, and the muscles [literally: body parts] are, muscles/body parts don't heal in the same way as before” |
|  |  | Maria | Self-employed | F | Small Workplace | “If you have something like, a hip broken and you wait to get to a hip surgery or a knee surgery so it affects terribly strongly like a person's, everyday life, so they might be like... what I'm thinking is that they would be the most fucked up of all these kinds of like, knee and hip problems.Then, of course, if there are some of like these endemic diseases such as diabetes or blood pressure, so they don't either like as such, affect your ability to function if they are like treated well. I guess exactly, musculoskeletal problems are, would be kind of... challenging.” |
|  |  | Kalevi | Employee | M | Small Workplace | “So well, yes these are also related, to that kind of health in part that just then, when I was diagnosed, type two diabetes, so and, and, and then this myocardial infarction, then they're kind of like that, of course, which have been put a little bit like things, a little bit different anyway, from a different perspective but uhm but that yes they are now also kind of related to that, as if, They're not like that they only come up at some point with things like that, and then they're also associated with that age even though they're actually related to health but uhm, but that when I was younger they didn't, didn't have to care like, in the same way, they didn't exist, hadn't existed per se . |
|  |  | Linnea | Self-employed | F | Bigger Workplace | “That's the worse side, but I do see like, seriously like, on the mental side, it's, like, so much better, to have some age, for a person than to be just about in your twenties; when the back gets stuck and, all the aches come. If at that point, especially if you've been in the same job all your life, it's a change, in a way, that you are facing, So, it certainly affects getting older in such a way that there will then be, someone might get depressed, well of course that affects you physically.” |
|  |  | Kerttu | Self-employed | F | Bigger Workplace | “Just as suddenly as possible, under the soil, probably someone like that... that, listens like hearing, finds that there is hearing loss a little, like lowered that it is not like, in some situation when there are a lot of people, around you don't like, children all talk something, then you can't concentrate that you don't when you're younger, you can't register, all the things around, around.” |
|  |  | Päivi | Employee | F | Small Workplace | “Of course, if you start to have some memory disorder, but you realize it, that, everything isn't right so, I guess there too you probably will feel yourself, old and sick” |
|  |  | Kalevi | Employee | M | Small Workplace | I notice, about myself well I've had one myocardial infarction and uhm, and, things like that obviously seem well I know I'm slower and, uhm, slower and stiffer than before and things like that you notice, but on the other hand I also notice that.. And then, then I also realize that I don't, maybe, yes, people's attitude has changed a little bit as I get older, |
|  |  | Tapio | Self-employed | M | Bigger Workplace | Health problems, well it is relevant so it's probably going to be the, essential part because this work is, is, quite physical at times too so. It's actually the most important factor, so that at that point if your physique can't take it then at that point you have to, at that point you have to change [hh], your genre [or: your sport], so then the company changes to something else |
| **2.** | **Meaningful moments** | Orvokki | Employee | F | Small Workplace | “We almost have that free time now that we're here, keeping this in order and, of course, taking care of it, and, of course, this kind of always comes with those extra expenses that We haven't really been able to travel now, but I hope that time will come” |
|  |  | Ilmari | Employee | M | Small Workplace | “The everyday life is maybe, probably, from Monday to Friday, weekends then, have then slightly different schedules, that's how I have imagined it.” |
|  |  | Kalevi | Employee | M | Small Workplace | “In everyday life that, I don't have anything so special... Not like maybe, strict daily routine but uhm but no, also such that, a little, then you can, live in a way... and to do things of my own, my own in music, for example, and other things that are not now, which hasn't been like that at the moment, but uhm, then there could be time, more time to do, all kinds of things.” |
|  |  | Viljami | Self-employed | M | Bigger Workplace | “Well... some of the good aspects are maybe that there will be, there will be experience and perspective and then, probably more of that time for yourself.” (Viljami, Male,56, Self-employed with employees) |
|  |  | Päivi | Employee | F | Small Workplace | “Well, I guess usually financially you start to have or should have, your debts paid off if you have had a house loan and... and of course the fact that there are no more these, there, at this age, the children have already flown out of the nest and... so these, these are what I kind of think of so that you are, free to do the thing, you don't have to anymore like, take care of someone.” |
|  |  | Päivi | Employee | F | Small Workplace | “Well, yeah, certainly, the travelling if I just can financially, then I would like to travel, and of course this, this island, going to the summer cottage, during summertime is, what I want to do as long as I can. I like to do handicrafts, read books, exercise as a hobby, cycling skiing, walking, gym; I do wish that I could do such things” (Päivi, Female,60, Employees in small workplace) |
|  |  | Olavi | Employee | M | Bigger Workplace | “Like, earlier, it has been so that earlier I have done civil disobedience research and been interested in a certain kind of activism so, so now we got money for it, and I have promised to write a book about it at the time, I had a dream, a dream of becoming a journalist and that, I could, as a journalist, as I was like interested in writing, and I was like, we were doing these small magazines, editorial work with friends back then, and we did interviews stories and took photos and everything, it was exciting, and I thought that, a journalist that's what I should become.” |
|  |  | Juhani | Self-employed | M | Small Workplace | “I have done my doctoral degree, and then I've written quite a lot. I don't know yet what I would do, nonfiction or fiction, or just for my own enjoyment, but writing is important to me. Some kind of physical hobby, something like yoga-style, will definitely stay with me all the time.” |
|  |  | Maria | Self-employed | F | Small Workplace | “I'd like to write a book, so this is how I kind of think, that then when I have retired or when I my reduce amount of work, so I'd like to write, an autofiction book.” |
|  |  | Kerttu | Self-employed | F | Bigger Workplace | “Three years come now so, in August, so, they had a good time, with the grandchildren and take a sleepover, and, and such a time, yes, such as that, I see in my eyes that hopefully, I will then have that I will still be healthy, that when I no longer have this company, that I will have more time to be with them that now I have, a few days off, but I spend them with them even now. So that the fact that I could give that help in such places, not as an actual nurse like every day, that when they go to work, but in such a way, among other things, help that when they need it once in a while or they, they go somewhere, to the village or, to some leisure time sometimes without a child, then come to sleepovers and things like that, I hope that I will have the strength to do so, even in my old age, and that they, the children, come to me, to us, that now it might be such a pleasant place.” |
|  |  | Olavi | Employee | M | Bigger Workplace | “Then, good aspects about it, well, of course, some good aspects, you can see how your offspring is growing up and, like the b- beginning of their lives and.., you can enjoy that.” |
|  |  | Linnea | Self-employed | F | Bigger Workplace | “I suspect that I might not be so much like into that, but it could be that, of course, if I had grandchildren so, then I would be with them.” |
|  | **Diminished Functionality** | Kerttu | Self-employed | F | Bigger Workplace | “Some things you sometimes notice that, well, now this doesn't go quite the same way as when I was younger, or Well, even if someone is exercising or somewhere, even while picking berries or something else that it's like bending over or someone or bicycle when I'm riding like that, jumping on a bicycle like that, before it was when you just jumped on it, now you have to kind of think about how that leg rises and, or when you're climbing a ladder like that, you kind of have to think, well, now, be careful that you don't just fall out of this, when before you didn't think so much about things like that if you climb something high or something like that, I just did it when I was younger, but that, now, I think a lot more about those kinds of safety issues.” |
|  |  | Viljam | Self-employed | M | Bigger Workplace | “Well, maybe now, lately, when I've been doing more sports, so, I notice that, no, the speed is not the same anymore as it used to be.” |
|  |  | Ilmari | Employee | M | Small Workplace | “It's also such a thing about your own cleanliness and like, like home and about doing hospitality that if I start to, like, accumulate dust or dirt or dishes, I notice that when I don't have the energy to take care of those things, then I'm probably old so that I can't anymore it's the, the maintaining of hospitality and this kind of a home, it's like a mental backbone so that it forces you to do and move and clean and, do things that aren't really nice, but you just have to do them so, this is also one thing that like if, things start to like to accumulate and pile up, then that's such a sign to me that, that if someone says to me that daddy could you clean up a little bit, so, I'm afraid t- then I'll be really old” |
|  |  |  |  |  |  |  |
|  | **Wisdom and Maturity** | Ilmari | Employee | M | Small Workplace | “I think it is a part of it that you like get that kind of calmness and there's not anymore like you realise that and, this kind of thing that, something like gardening or taking care of greenery or you can, really start to think about it. It was burnout, and it's, and that's how I, recognise if I start to have stressful tasks so then I can reflect pretty quickly that now, stop, or like I know this is a path that leads to destruction for everyone, or, for colleagues, so now I may be like know more about how to, dare more to take more time for myself so maybe that's part of getting older.” |
|  |  | Juhani | Self-employed | M | Small Workplace | “Somehow, learn to appreciate life..you learn to maybe enjoy life more, in such a way that you're not, you're not constantly amassing more, for example, new experiences, you realize that those experiences, those everyday experiences can be valuable and, and of course the fact that, experience, professional experience, for example, does bring this kind of, depth and, professional skill, so in a way, if you think about professional life and, these are kind of like clichés but it's so that, so that if you've seen like, seen so many, many kinds of life, then maybe you participate- can participate- you can like .. somehow, more objectively, deal with the things that life brings.”(Juhani , Male, 59, Solo-Self-employed) |
|  |  | Kalevi | Employee | M | Small Workplace | “Well, in some ways, yes that, yes I do; I believe that a person develops, over the course of his life and, if that's right, thinks so, yes then, for sure, there should be such wisdom in that old age already” |
|  |  | Maria | Self-employed | F | Small Workplace | “Maybe this kind of self-confidence comes so that surely then, I mean life experience brings this kind of self-confidence, at work, you can think that you don't need, so that of course, as an entrepreneur, there is not that much of it since nobody is, like as an entrepreneur now nobody is bossing me around in any way but, I get to be like my own boss [literally: my own lord], but that, at work there will be like this that can like say, directly that this thing is not okay for me, and, that you have this kind of self-confidence in work-, working life, and then self-confidence like outside of working life as well so that, it doesn't matter what people think ..what I do or, how I dress or what I am so that it's all the same, this kind of, this kind of like easiness, in that sense.” |
|  |  | Emilia | Employee | F | Bigger Workplace | “The fact that understanding is increasing all the time, that one's self-esteem improves, the fact that you no longer care about what other people think.” |
|  | **Defiance of stereotypes** | Emilia | Employee | F | Bigger Workplace | “Maybe it's just like I said that I feel like I still have the soul of a forty-year-old inside this old body, so, so I notice my age, only from this body. From the fact that I'm no longer, I can no longer do the things I did 20 years ago or 40 years ago, and that's kind of annoying in a way. |
|  |  | Kalevi | Employee | M | Small Workplace | “Well, I see that I, I'm older than before, but I don't uhm, I don't consider myself to be particularly old, me, I feel like it's kind of like I've never, uh, I'm always just the age I am, and then, other people are older versus older or younger. I have, I don't have that big... I don't, I don't think so much about age; for example, most of my colleagues are, at the moment, my children's age. But I don't feel like I'm old, but I do notice things like that. I notice signs of ageing, so this is natural, but I still don't feel old.” |
|  |  | Sofia | Employee | F | Bigger Workplace | “I would never want to be such a grumpy old granny. I mean, it was like, every time someone asked me my age, I really had to start calculating, a minute, wait a minute, [laughs] that I was stuck there at 20 and 21 so that I'm always 20 and 21”. |
|  |  | Tapio | Self-employed | M | Bigger Workplace | “Well, I wouldn't want to age, of course, but evolution does its job.” |
|  |  | Maria | Self-employed | F | Small Workplace | “So these were my thoughts, and then I've kind of thought about it too that, because I, feel, being like, really like young, regarding my mind, for me, it's, terribly hard to think that I am, over sixty, I feel like I'm young and I am, healthy, and in good shape so maybe I'm fooling myself a little bit too by thinking that, well, that I'm actually only fifty.” |
|  | **Proximity to death** | Emilia | Employee | F | Bigger Workplace | “So, in a way that mortality comes here, starts to get close at this age because you start to have those colleagues, who have gone [died] because of some seizure, and left the crowd I had just, a class reunion over the weekend, of our sixty, who graduated so, thirty were there. And two of us have departed from time to eternity, from these sixty.” |
|  |  | Kalevi | Employee | M | Small Workplace | “Well, exactly, just these kinds of things that, of course, that well, the fact that when you have had your first myocardial infarction, it puts a little bit of a new frame when you were about to die from it.” |
|  |  | Olavi | Employee | M | Bigger Workplace | “ I don't; there is some concept for it, I just don't remember it since my Latin is so bad, but I've thought sometimes that, like, people too, like, it probably comes often there somewhere around 50, or at least these conversations happen somewhere around age 50, like, with people, so then in a way, uhm, inevitably, a person starts to recognise that death is now.. closer than it has been, sometime in the past and people in their twenties and thirties, in my opinion, don't need to start thinking about death yet let alone a 12-year-old. But it's true that, like, a fifty-year-old needs to start thinking about a little bit already. And in a way, it's like, when life gets to that point where you slowly start, like little things first, first probably without you realising it, and you start preparing for that death. Maybe that's what old age is about.” |
|  |  | Maria | Self-employed | F | Small Workplace | “Physically deteriorates, and kind of like, well, cognitively and memory, all those kinds of things work like a little slower and, truly death is coming closer, it is, it is a fact, and it's, just awful.” (Maria, female 61, Solo self-employed) |
|  |  | Juhani | Self-employed | M | Small Workplace | “Personal and difficult subject of course, because getting older means that, that death, is approaching and, none of us wants to, that much, to think about their own death.” (Juhani , Male, 59, Solo-Self-employed) |
